# Acquired ABC-transporter overexpression in cancer cells: transcriptional induction or Darwinian selection?

**DOI:** 10.1007/s00210-021-02112-3

**Published:** 2021-07-08

**Authors:** Dirk Theile, Pauline Wizgall

**Affiliations:** Department of Clinical Pharmacology and Pharmacoepidemiology, University of Heidelberg, Im Neuenheimer Feld 410, 69120 Heidelberg, Germany

**Keywords:** Multidrug resistance, ATP-binding cassette transporters, P-glycoprotein, Pregnane-x-receptor, Induction, Selection

## Abstract

Acquired multidrug resistance (MDR) in tumor diseases has repeatedly been associated with overexpression of ATP-binding cassette transporters (ABC-transporters) such as P-glycoprotein. Both in vitro and in vivo data suggest that these efflux transporters can cause MDR, albeit its actual relevance for clinical chemotherapy unresponsiveness remains uncertain. The overexpression can experimentally be achieved by exposure of tumor cells to cytotoxic drugs. For simplification, the drug-mediated transporter overexpression can be attributed to two opposite mechanisms: First, increased transcription of ABC-transporter genes mediated by nuclear receptors sensing the respective compound. Second, Darwinian selection of sub-clones intrinsically overexpressing drug transporters being capable of extruding the respective drug. To date, there is no definite data indicating which mechanism truly applies or whether there are circumstances promoting either mode of action. This review summarizes experimental evidence for both theories, suggests an algorithm discriminating between these two modes, and finally points out future experimental approaches of research to answer this basic question in cancer pharmacology.

## Introduction

Chemotherapy remains one of the most frequently delivered therapy approaches to patients with tumor diseases. However, the development of multidrug resistance (MDR) limits the efficacy of chemotherapeutic agents and therefore hampers effective cancer treatment (Longley and Johnston 2005). Several mechanisms and molecular alterations in cellular pathways are associated with the development of the complex cellular process of MDR. For instance, alterations of drug targets, enhanced drug metabolism (detoxification), sustained growth stimulation (pro-survival signaling), changes of macroscopic cancer physiology (tissue perfusion, behavior of neighboring cells, and immune cell responses), improved DNA damage repair, cell cycle arrest, and apoptosis resistance have been identified to be implicated in MDR (Longley and Johnston 2005; Gottesman et al. 2016). Moreover, ATP-binding cassette (ABC-) transporters, drug-metabolizing enzymes (DMEs), and nuclear receptors (NRs) are thought to contribute to MDR. Physiologically, these proteins are essential parts of a general xenobiotic defense machinery, that recognizes, metabolizes, and eventually disposes a large variety of compounds. This results in the protection of the single cell or the whole organism from harmful noxes, but also modulates kinetics of therapeutic drugs (Sarkadi et al. 2006; Amawi et al. 2019).

### Relevance of ABC-transporters for MDR

ABC-transporters have been identified to be involved in mediating target-nonspecific MDR (Gottesman et al. 2002). By using the energy of ATP hydrolysis, these large transmembrane glycoproteins serve as penetration modulators of a large variety of structurally unrelated compounds. Overexpression of this family of ATP-dependent “drug pumps” has been implicated in the development of MDR. Their true relevance for (acquired) clinical unresponsiveness to chemotherapy remains controversial (Tamaki et al. 2011; Robey et al. 2018; Borst 2020). However, ABC-transporters can mediate considerable MDR in vitro or in animal models, likely by decreasing the uptake or accumulation of chemotherapeutic agents in cancer cells (Szakács et al. 2006; Robey et al. 2018). Out of 48 known human ABC-transport proteins, about two dozen of them have been associated with MDR (Dean and Annilo 2005; Ween et al. 2015): Most well evaluated is P-glycoprotein (P-gp), encoded by *ABCB1*. P-gp has been investigated for almost five decades and remains the most important MDR-related ABC-transporter (Lee et al. 2010; Robey et al. 2018). The breast cancer resistance protein (BCRP, encoded by *ABCG2*) is in fact structurally different from P-gp but shows a comparably broad substrate specificity that partly overlaps with P-gp (Lee et al. 2010). Eventually, the ABC-transporters of the C subfamily (encoding the MDR-related proteins) are also known to mediate MDR. However, exposure of cancer cells to cytotoxic drugs has been demonstrated to cause overexpression of a variety of other ABC-transporters (Szakács et al. 2006).

Although DMEs (e.g., cytochrome P-450 isoenzyme 3A4) (García-Martín et al. 2006; Robertson et al. 2008; Tian and Hu 2014) or uptake transporters of the solute carrier family (Li and Shu 2014) have been recognized to contribute to MDR, this review article will solely focus on ABC-transporters, being the classical mediators of MDR.

### Relevance of nuclear receptors for MDR

Acquired MDR can result from induction of transporter genes such as *ABCB1*. Many molecular pathways have been described that cause enhanced transcription of *ABCB1.* For instance, alteration of chromatin structure, gene arrangement, promoter demethylation, or histone acetylation are known to occur during malign transformation or upon drug treatment (Chen and Sikic 2012). These alterations are thought to sustain once they are installed in their respective cellular context or chromatin status. In contrast, drug-regulated NRs can dynamically modulate MDR by fine-tuning the expression of their respective target genes, including many DMEs, drug-conjugating enzymes, and drug transporters involved in pharmacokinetics and MDR (Chen et al. 2012; Evans and Mangelsdorf 2014).

Generally, the nuclear receptor super family consists of many types of receptors that are divided into four subfamilies, depending on their function (e.g., homo-dimerization) or mechanism (e.g., ligand dependent) (Mangelsdorf et al. 1995). One of those subfamilies consists of ligand-dependent NRs, that migrate into the nucleus after binding endogenous or exogenous compounds. There, they bind to their receptor-specific xenobiotic response elements or hormone response elements at the promoter regions of their specific target genes (Tukey and Strassburg 2000; Mangelsdorf et al. 1995; Evans and Mangelsdorf 2014). The pregnane-x-receptor (PXR) is probably the most prominent NR known to induce MDR (Chen and Nie 2009; Rigalli et al. 2019). It binds a wide variety of endogenous or exogenous compounds such as hormones, drugs, food additives, and pollutants. After ligand binding, PXR coordinates the expression of many CYPs and UGTs, as well as MDR-related drug transporters including *ABCB1*/P-gp and *ABCC2*/MRP2 (Bertilsson et al. 1998; Blumberg and Evans 1998; Rigalli et al. 2019).

PXR shares a lot of target genes with the constitutive androstane receptor (CAR), that is also widely accepted to be involved in the induction of MDR (Rigalli et al. 2019). However, in contrast to PXR, CAR does not necessarily require ligand-binding to become active but rather needs cross-talk with other NRs (e.g., PXR) for xenobiotic response (Kast et al. 2002; Xie et al. 2000). Another receptor that resembles PXR and CAR characteristics and even cross-talks with those, too, is the aryl hydrocarbon receptor (AhR). AhR also tunes the expression of phase I–III proteins including CYP1A1, CYP1A2, and *ABCG2*/BCRP (Patel et al. 2007; Rigalli et al. 2019).

## Transcriptional induction or Darwinian selection?

Research in the field of MDR and ABC-transporter overexpression has been extensive. Although numerous molecules or regulatory pathways have been identified (Alexa-Stratulat et al. 2019), the exact or decisive mechanism of acquired ABC-transporter overexpression could not yet be fully explained. A wide variety of experimental set-ups using different exposure times or drug concentrations have been used in vitro to cause ABC-transporter overexpression and phenotypical MDR. Besides the many mechanisms leading to transporter overexpression (Chen and Sikic 2012; Yano et al. 2018), a simplification is made to promote the comprehensiveness of the two opposing mechanisms presented here. In this theoretical framework, development of MDR transporter overexpression and functional MDR can be caused by the two following modes: First, increased transcription of ABC-transporter genes mediated by drug-activated nuclear receptors (e.g., PXR) can lead to transporter overexpression. Second, cytotoxic drug–mediated Darwinian selection of sub-clones intrinsically overexpressing ABC-transporter genes/proteins (e.g., through gene amplification (Huff et al. 2005, 2006; Gerlinger and Swanton 2010)). Certainly, this is not a black-or-white scenario. Instead, there shall be circumstances when both scenarios act on cancer cells concurrently. For instance, when the selective pressure is rather mild (drug concentrations considerably below the IC_50_ of sensitive cells), proliferation of cells that have adapted on a single-cell level is promoted (transcriptional induction) (Chisholm et al. 2015; Pisco et al. 2013). To date, there however is no definite data indicating which mechanism applies (in vitro or in vivo) or whether there are circumstances promoting either mechanism.

This review summarizes existing experimental evidence for NR-mediated transcriptional induction vs. selection mechanisms leading to ABC-transporter overexpression and MDR in cancer cells. Finally, experimental set-ups will be suggested, which might facilitate further understanding of the development of ABC-transporter-mediated MDR. Such experimental data can potentially help to improve clinical treatment regimens against cancer.

### An algorithm distinguishing transcriptional induction from Darwinian selection

**Fig. 1:**
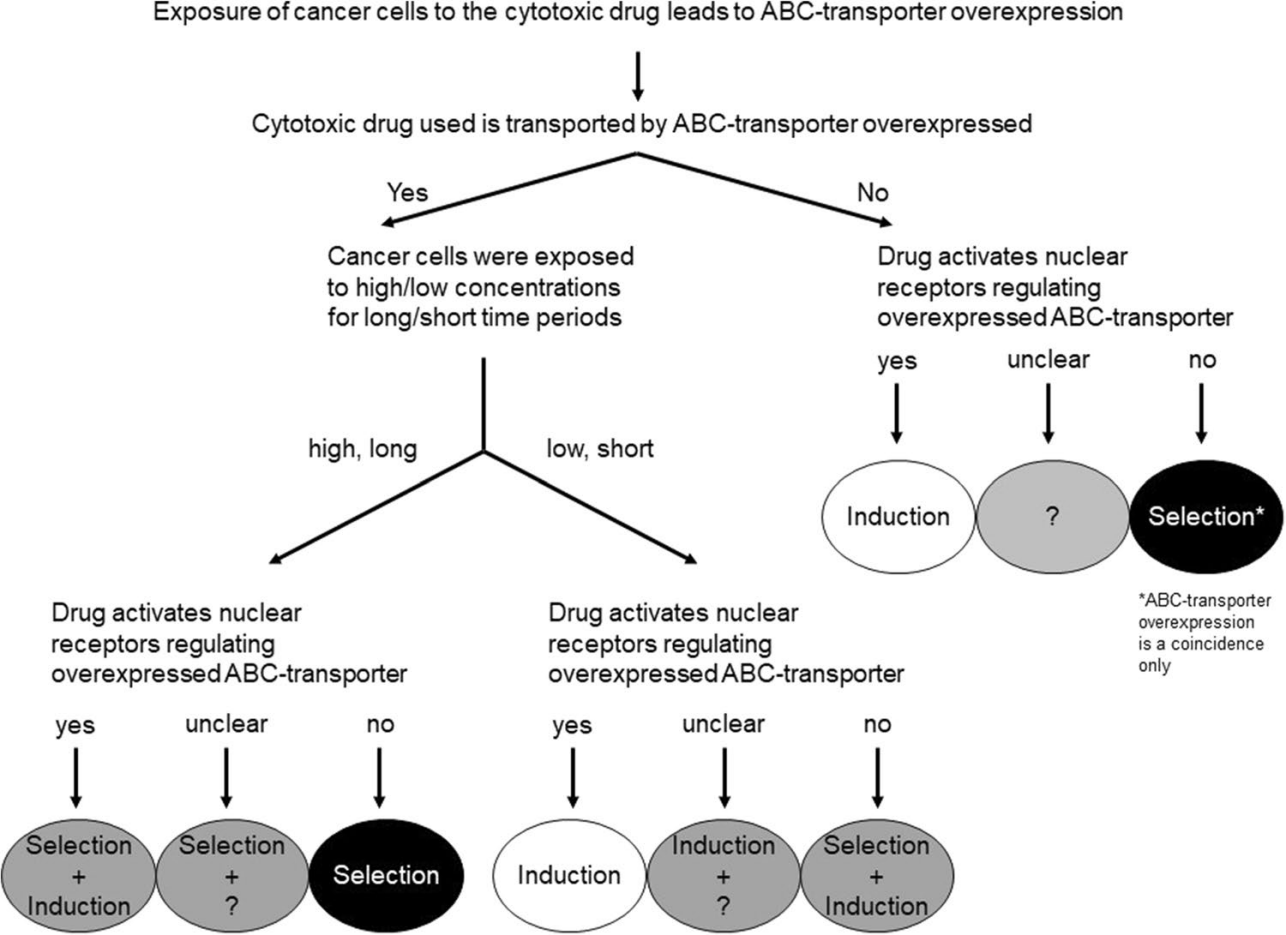
An algorithm proposed to distinguish the simplified regulatory origin of drug-mediated ABC-transporter overexpression. If exposure of cancer cells to a cytotoxic drug led to the overexpression of an ABC-transporter, some decisive questions should be stated. Their answers can guide the estimation whether ABC-transporter overexpression resulted from transcriptional induction (e.g., short drug exposure to low concentrations of a drug known to activate important nuclear receptors) vs. Darwinian selection (e.g., long-term exposure to high concentrations). In some circumstances, ABC-transporter overexpression only is a coincidence (epiphenomenon) and multidrug resistance actually resulted from non-transporter mechanisms

Integrating the huge amount of data from experiments evaluating acquired ABC-transporter overexpression, we here suggest a simplified algorithm. With the help of this algorithm, NR-mediated transcriptional induction is distinguished from Darwinian selection (Fig. 1). After exposing cancer cells to a distinct cytotoxic drug, the cell population subsequently shows enhanced expression of ABC-transporters. The following questions and their answers can then direct to a more precise estimation of the actual mode of ABC-transporter overexpression:

1. Is the cytotoxic drug used transported by the overexpressed ABC-transporter?
  -For instance, when the cytotoxic drug treatment led to the overexpression of an irrelevant ABC-transporter (not extruding the compound of interest), this overexpression was most likely not advantageous to the cell. This means Darwinian selection was unlikely involved in ABC-transporter overexpression. If the cytotoxic drug can activate a NR that regulates the expression of this overexpressed transporter, induction might in fact have taken place, but without MDR relevance. In contrast, if the drug does not activate a NR being responsible for the expression of the ABC-transporter overexpressed, selection might be implicated in drug resistance, but the transporter overexpression only is a coincidence. The actual drug resistance is rather mediated by non-transporter mechanisms (e.g., target downregulation, DNA damage repair).
    Example: Exposure of cancer cells to cisplatin can increase the expression of ABCG2/BCRP (Vesel et al. 2017). However, cisplatin is not transported by BCRP (Yuan et al. 2009) and it does not activate the main ABCG2 regulator AhR (Sasaki-Kudoh et al. 2018). In consequence, ABCG2/BCRP overexpression upon cisplatin treatment likely is a non-related coincidence.
  -On the other hand, exposure of cancer cells to cytotoxic drugs can enhance the expression of ABC-transporters that can facilitate the drug’s efflux from the cells, thus protecting the cell from the harmful effect (“Yes,” overexpressed ABC-transporter extrudes the compound of interest).
    Example: Cisplatin treatment increases the expression of ABCC2/MRP2 (Schrenk et al. 2001; Demeule et al. 1999), an ABC-transporter known to mediate cisplatin resistance through extruding cisplatin (or its glutathione conjugate) out of the cell (Cui et al. 1999; Guminski et al. 2006).
2. What was the treatment mode that led to the overexpression of the ABC-transporter (being capable of transporting the respective drug)?
  -If the cells were exposed to concentrations being considerably lower than pre-treatment IC_50_ for a short period of time (e.g., 1–3 days), transcriptional induction is a very probable scenario. This is especially relevant when the drug is known to activate NRs that regulate the expression of the ABC-transporter overexpressed. In contrast, when the cytotoxic drug does not interfere with known regulatory NRs, induction (by other mechanisms) cannot be excluded, but Darwinian selection likely contributed to the ABC-transporter overexpression.
    Example: Low concentrations of paclitaxel increase the expression of the paclitaxel transporter ABCB1/P-gp (Schöndorf et al. 2003; Theile et al. 2011), likely through activation of PXR, a major regulator of ABCB1/P-gp expression and induction (Harmsen et al. 2010).
  -In contrast, when cells were treated with high cytotoxic drug concentrations (killing the majority of sensitive cells, e.g., IC90) for a prolonged period of time (or repetitive cycles of exposure), Darwinian selection of sub-clones with intrinsically overexpressed ABC-transporters seems very nearby. This is of even higher likelihood, when the cytotoxic drug does not activate important NRs (e.g., PXR, CAR, or AhR) known to regulate the expression of the ABC-transporter overexpressed. In fact, when the cytotoxic drug can activate those NRs, transcriptional induction of transporter genes might play a minor role, but Darwinian selection should remain the most important mode given the high selective pressure (IC90).
    Example: Long-term exposure of cells to 5-FU increases the expression levels of ABCG2/BCRP (Yokoo et al. 2007) and ABCC5/MRP5 (Hagmann et al. 2009). Although both ABC-transporters have been implicated in 5-FU resistance through cellular efflux (Yuan et al. 2009; Pratt et al. 2005), 5-FU is not known to functionally interfere with major NRs regulating ABCG2/BCRP or ABCC5/MRP5 expression (e.g., AhR or PXR). In consequence, transcriptional induction is of minor relevance. In contrast, cells with overexpressed ABCG2/BCRP or ABCC5/MRP5 withstood the selective pressure and became selected.

When the scientific literature about acquired ABC-transporter overexpression or MDR is screened for experimental set-ups, some publications can well be assigned to transcriptional induction (Chen et al. 2009; Harmsen et al. 2010; Theile et al. 2011) or Darwinian selection (Abbadessa et al. 1992; Huang et al. 2006; Mensah-Osman et al. 2007; Slapak et al. 1990, 1994). This advocates for the appropriateness of the suggested algorithm (Fig. 1). Representative articles are highlighted in Table 1. Two of them shall be explained and discussed in more detail:

**Table 1:** Examples of experimental studies representing Darwinian selection or transcriptional induction modes leading to overexpression of ABC-transporters and MDR

Citation	Cell line	Drug	Concentration	Exposure time	Gene	Outcome	Comment
Examples for Darwinian selection							
Abbadessa et al. (1992)	Friend leukemia	Daunorubicin	High (*not specified*)	Repetitive24 h cycles	*ABCB1*	High resistance	Resistance not reversible by verapamil, suggesting other mechanisms being implicated in the phenotype
Huang et al. (2006)	LS174T (high PXR), Caco-2 (low PXR), A549 (low PXR)	Vincristine	10 nM, 100 nM (IC_50_ = 2 nM for LS174T cells; IC_50_ for other cell lines not quantifiable)	24, 48, 72 h	*ABCB1*, *ABCC1*, *ABCC2*, *ABCC3*	LS174T: *ABCB1* 3–fourfold increase; Other cell lines irrelevant increases	Increase in *ABCB1* expression in LS1274T cells did not lead to lowered vincristine uptake, suggesting that *ABCB1*/P-gp overexpression is a non-related coincidence
Mensah-Osman et al. (2007)	OS187	Etoposide	50 µM (about IC_50_)	48 h	*ABCB1*, *CYP3A4*	*ABCB1*: fourfold enhancement; *CYP3A4*: 30-fold enhancement	Compared to etoposide, rifampicin (a PXR activator) had lower effects in OS187 cells, suggesting that transcriptional induction is of minor relevance
Slapak et al. (1990)	PC4, C7D	Doxorubicin	5–160 ng/ml	10 cell passages	*ABCB1*	Doxorubicin resistance occurred after prolonged exposure times; *ABCB1* overexpression occurred with higher doxorubicin (> 80 ng/ml) concentrations	Selection processes promote non-transporter-mediated MDR phenotypes
Slapak et al. (1994)	PC4, C7D	Vincristine	5–160 ng/ml	10 cell passages	*ABCB1*, *ABCC1*	*ABCB1* overexpression was only achieved with high a concentration used for prolonged times	
Examples for transcriptional induction							
Chen et al. (2009)	MDA-MB-231; MCF-7	SR12813 (PXR activator)	0.2 µM (SR12813 was not cytotoxic up to 1 µM)	Up to 72 h	*ABCB1*, *CYP3A4*	Strongest *ABCB1* induction (about 4–fivefold) after 8 h; decline afterwards; paclitaxel/tamoxifen resistance occurred towards low concentrations, but not high concentrations	Given the non-cytotoxicity of 0.2 µM, selection is largely ruled out; PXR activation and thus *ABCB1* induction however seems to only mediate resistance to rather low cytotoxic drug concentrations
Harmsen et al. (2010)	LS180	Rifampicin (PXR activator)	10 µM	48 h	*ABCB1*	Rifampicin treatment lowered doxorubicin uptake by 30% and cytotoxic potency by 40%	
Theile et al. (2011)	HNO (head and neck cancer cell lines)	Paclitaxel	Respective IC_20_	72 h	Several ABC-transporters including *ABCB1*	Paclitaxel increased mRNA levels 2–fourfold, being partly accompanied by enhanced drug resistance	Effects on mRNA levels and cytotoxic potency of anti-cancer drugs were rather small. The mechanistic link had not been evaluated

#### Transcriptional induction and subsequent MDR

In their work, Chen and co-workers (Chen et al. 2009) aimed to determine the role of PXR during the development of drug resistance in breast cancer cells.

To ensure selective PXR activation without anti-proliferative off-target effects, a synthetic PXR ligand was used called SR12813. Treatment of MCF-7 and MDA-MB-231 cells (co-transfected with a PXR overexpression plasmid) for 24 h enhanced PXR activity concentration-dependently, peaking at about 0.2 µM. Moreover, mRNA levels of *CYP3A4* and *ABCB1* became accordingly induced after exposure to 0.2 µM. Interestingly, the mRNA expression levels peaked after 8–12 h of continuous exposure but declined again afterwards. To eventually assign PXR activation by SR12813 treatment to cytotoxic drug resistance, breast cancer cell lines were initially pre-treated with 0.2 µM SR12813 for 12 h before subjecting them to drug resistance assays. The results clearly showed that PXR activation rendered the cancer cells resistant to low paclitaxel concentrations (20 nM, 50 nM) but did not change sensitivity towards high paclitaxel concentrations (500 nM, 1000 nM). Moreover, there was only about 10% difference of viability between SR12813- and DMSO-treated cells. Nevertheless, the fact that the provoked resistance in response to SR12813 was less pronounced in MDA-MB-231 cells (low PXR expression) compared to MCF-7 cells (high PXR expression) represents additional proof for the NR to be directly involved in the development of drug resistance. Because SR12813 was not toxic to the cells, concurrent selection processes can be largely excluded.

In summary, the experiments performed by Chen and co-workers elegantly showed that drug-activated PXR can enhance the expression of ABC-transporters, leading to drug resistance in cancer cells. However, the data strongly suggests that NRs only partly contribute to the MDR phenotype by mediating short-term transcriptional inductions of respective genes and resistance to rather low concentrations of cytotoxic drugs.

#### Darwinian selection and subsequent MDR

The scientific publication by Hembruff and co-workers (Hembruff et al. 2008) was one of the first providing detailed insights into the development of MCF-7 cells’ resistance against several cytotoxic drugs, including paclitaxel. MCF-7 cells were made paclitaxel resistant through a step-up approach: Starting 1/1000-fold below the pre-treatment IC_50_ (IC_50_: 0.56 nM; first selection concentration: 0.56 pM), paclitaxel concentrations were increased every 2 weeks until 99 nM. However, detectable resistance and enhancement of *ABCB1* mRNA expression only occurred above a certain concentration (“selection dose”) of 3.66 nM (6.5-fold higher than initial IC_50_). This means high concentrations selected for sub-populations that were intrinsically paclitaxel resistant, partly mediated by the overexpression of ABC-transporters such as *ABCB1*/P-gp. Moreover, the mentioned selection concentration was not only needed to enhance expression levels or phenotypic paclitaxel resistance but also was the first selection step leading to lowered paclitaxel uptake into the cells. Interestingly, no linear relationship between drug resistance and drug accumulation could be observed in this context: With the onset of phenotypic paclitaxel resistance and *ABCB1*/P-gp overexpression, drug accumulation was in fact reduced by about 20% compared to parental MCF-7 cells, but higher degrees of paclitaxel resistance were not accordingly accompanied by proportional reductions of paclitaxel uptake. This indicates that additional, drug uptake-independent mechanisms are responsible for the development of further enhanced drug resistance. Long-term MDR might be mediated by, e.g., apoptotic resistance or other advantageous traits surviving cells had been selected for. This hypothesis is also undermined by the fact that changes in drug uptake and sensitivity towards paclitaxel could not be fully reversed by cyclosporin A, a pan ABC-transporter inhibitor.

In summary, Hembruff et al. yielded MDR cells, that have been selected in a Darwinian manner for their elevated ABC-transporter expression but also transporter-independent mechanisms of MDR. Moreover, while overexpression of ABC-transporters and thus reduced drug uptake likely contributes to low levels of drug resistance, transporter-independent mechanisms mediate the high degrees of chemotherapeutic drug resistance.

## Experimental set-up to distinguish transcriptional induction from Darwinian selection in vitro

**Fig. 2:**
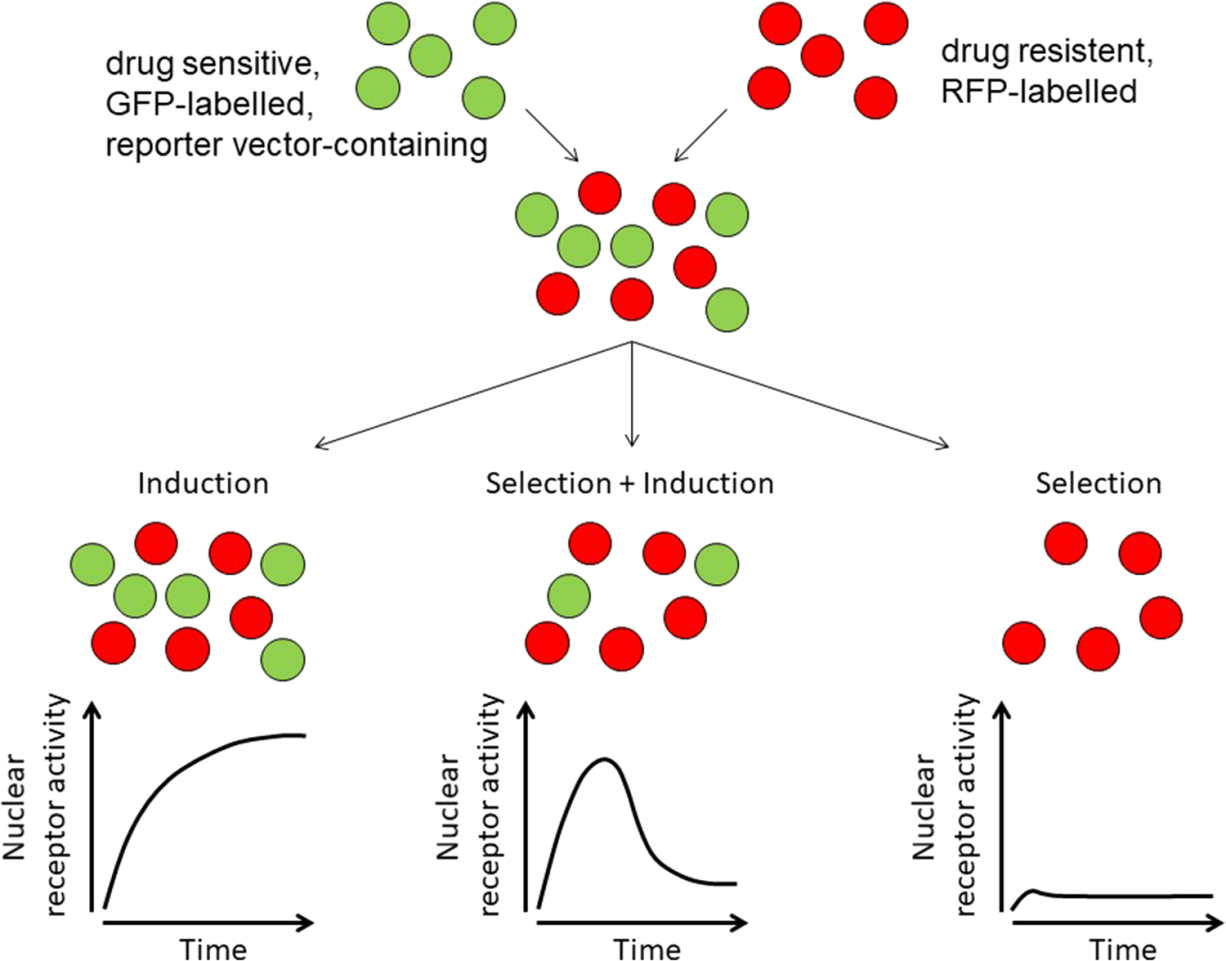
Graphical depiction of an experimental approach to distinguish nuclear receptor–mediated transcriptional induction vs. Darwinian selection in vitro. After generating two sub-cell lines (drug resistant, red fluorescent protein (RFP-) labelled; drug sensitive, green-fluorescent protein (GFP-) labelled with additional reporter gene under the control of a nuclear receptor regulating the ABC-transporter of interest, e.g., *ABCB1*/P-gp), a mixture of both populations (e.g., 1:1 ratio) is exposed to the cytotoxic drug of interest. Concurrent recording of reporter signal and GFP/RFP fluorescence (quantitative composition of the cell population) over time can eventually estimate the origin of cytotoxic drug–mediated ABC-transporter overexpression: (a) transcriptional induction, (b) concerted transcriptional induction and Darwinian selection processes, (c) Darwinian selection. See text for details

To date, no comprehensive research directly juxtaposing transcriptional induction vs. Darwinian selection during the development of ABC-transporter-mediated MDR has been performed. To clearly distinguish the two different scenarios suggested here and to retrace the decisive criteria displayed in Fig. 1, experimental insights are necessary. One possible approach will be explained in the following (Fig. 2).

In preparation, two stably transfected, easy-to-distinguish sub-cell lines need to be generated:

Initially, cells are stably transfected (or transduced) with a vector encoding for a marker that can be used to track, monitor, and quantify the number of cells of this population. For instance, using a vector for red fluorescent protein (RFP) generates a population of cells with high red fluorescence, subsequently called the “red” cell population. This “red” cell line is then turned drug resistant by step-wise increase of cytotoxic drug concentrations in the growth medium as outlined earlier.

On the other hand, a “green” sub-cell line is generated through stable expression of the green-fluorescent protein (GFP). In addition, this cell line is made expressing another marker being regulated by the NR of interest. For instance, luciferase-based assays to monitor PXR activity in cancer cells have previously been used by us (Rigalli et al. 2012, 2013, 2018) and others (Harmsen et al. 2010; Basseville et al. 2011; Masuyama et al. 2007). Importantly, the two generated cell lines (“red” and “green”) ideally exhibit the same proliferation rates. Otherwise, one cell line will ultimately outgrow the other, potentially flawing the results and leading to misinterpretations.

The two cell lines (distinguishable by flow cytometry for RFP and GFP) can then be co-cultured (e.g., 1:1 ratio) and subjected to treatment with the cytotoxic drug of interest. Close evaluation of NR activity and cell numbers of the “green” population compared to the “red” population will then allow to distinguish between drug-induced enhanced transcription of ABC-transporter genes, Darwinian selection of “fitter” cells, or a blend of both mechanisms (Fig. 2).

Example of scenarios: If (a) cell numbers of the “green” and the “red” populations stay the same throughout drug treatment and NR activity is considerably increased in parallel, strong and MDR-relevant transcriptional adaptation likely occurred in the “green” cells, now being resistant. In contrast, if (c) only “red” cells survived the drug treatment and no relevant NR activity was observed in “green” cells at the beginning of the drug treatment, the surviving “red” cells have been selected in a Darwinian manner due to their advantageous, resistant phenotype. However, if (b) “green” cell numbers decrease over time compared to “red” cell numbers and enhanced NR activity was recorded during the onset of resistance, both selection of “red” cells and initial transcription-mediated defense against the cytotoxic insult in “green” cells occurred.

With varying treatment concentrations of different drugs (PXR activators vs. non-activators) or different cell culture mixtures (e.g., 1:10 “green”/ “red” ratio), a huge experimental variety can be achieved.

## In vivo reporter gene assays to monitor transcriptional induction in animal tumor models

The limited knowledge on the relevance and regulation of ABC-transporters in chemotherapy-resistant tumors is due to lacking clinical trials prospectively evaluating pre- and post-chemotherapeutic expression levels and associating them with intratumoral drug concentrations and parameters of response. Despite their theoretic feasibility, such studies again cannot scrutinize the mechanistic tropism of iatrogenic ABC-transporter overexpression. Hence, meaningful animal models are needed. A well-characterized model is the mouse, having two homologous genes (*mdr1a* and *mdr1b)* that are very well comparable to human *ABCB1*/P-gp structure and function (Croop et al. 1989). *mdr1a*’s regulatory elements (promoter) share 70% nucleotide sequence identity and are recognized by murine nuclear factors being similar to human PXR. Taken together, mouse *mdr1a* gene expression is an excellent surrogate to monitor P-gp regulation in an in vivo setting.

**Fig. 3:**
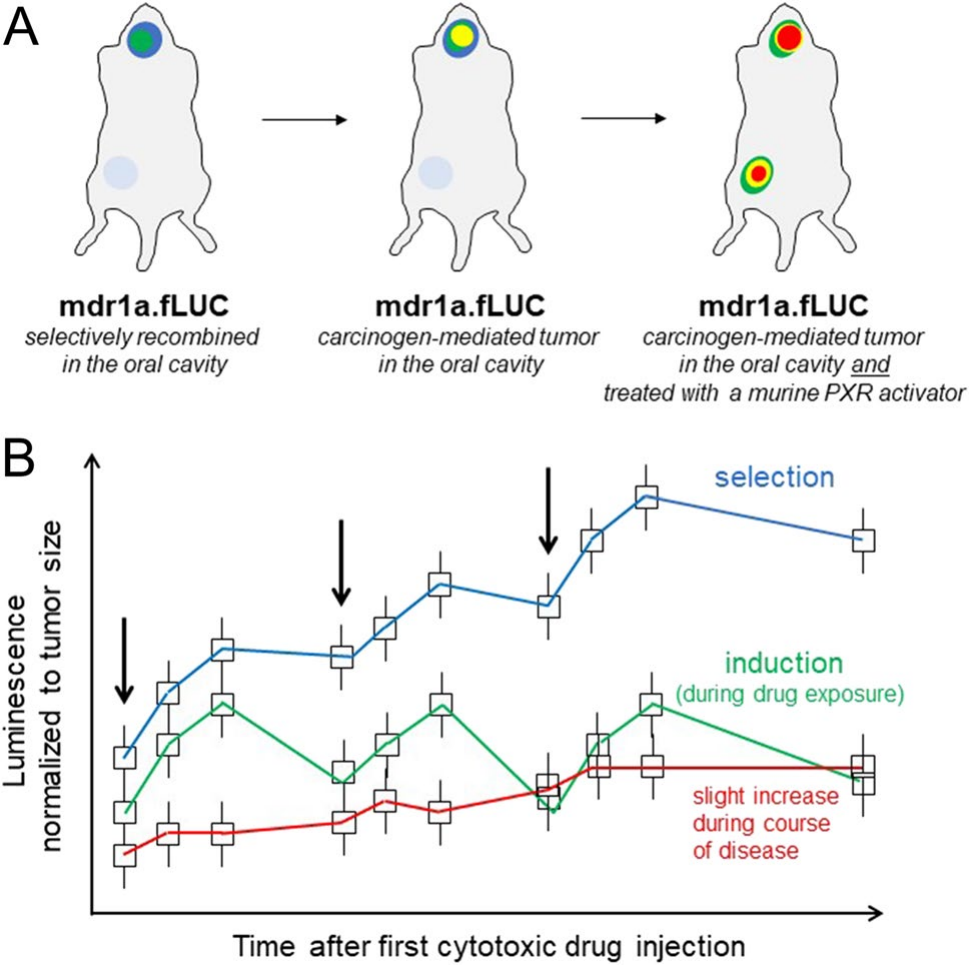
In vivo reporter gene assay approach to distinguish nuclear receptor–mediated transcriptional induction vs. Darwinian selection leading to *mdr1a* overexpression in murine cancer cells. (A) Bioluminescence images of mdr1a.fLUC mice with Cre*PR1-mediated recombination in the oral cavity (left), tumoral increase of bioluminescence (representing *mdr1a* transcriptional induction) during carcinogenesis in carcinogen-treated mdr1a.fLUC mice (middle), and drug-induced enhancement of bioluminescence during treatment with a murine PXR activator (e.g., taxane). Note the slight intestinal background bioluminescence in untreated animals (left, middle) and the strong intestinal luminescence in anti-cancer drug-treated mice. This off-tumor luminescence can be used as a control verifying sufficient systemic exposure to the murine PXR-activating anti-cancer drug (e.g., taxane). (B) Idealized kinetics of bioluminescence values (exemplary mean values ± S.D.) from mdr1a.fLUC mice with tumors in the oral cavity. Luminescence is normalized to tumor size (e.g., square inch of 2D pictures) and represents enhancement of *mdr1a* expression during natural course of disease or transcriptional induction (return to baseline between cycles of drug administration) vs. Darwinian selection (constant increase of *mdr1a* expression, carrying over through washout phase after last drug administration) of *mdr1a* overexpressing cancer cells. Arrows indicate administration of a potentially murine PXR-activating anti-cancer drug (e.g., taxane)

To monitor *mdr1a* expression in real time during taxane exposure, Gu and co-workers had replaced one of the two *mdr1a* alleles by the firefly luciferase (fLUC) gene through homologous recombination: mdr1a.flox mice were used as targets for Cre-mediated recombination to establish mdr1a.fLUC mice with fLUC expression under the control of the endogenous *mdr1a* gene locus (Gu et al. 2009). Consequently, throughout the mouse body, one *mdr1a* allel was replaced by the fLUC gene. This in turn meant that luminescence was well detectable in tissues physiologically expressing high levels of *mdr1a* such as the intestine and liver. Moreover, inducibility of luminescence during taxane exposure or return to basal luminescence values on drug withdrawal has been verified later on (Gu et al. 2013). Together, this murine in vivo reporter gene approach can be used to study NR-mediated transcriptional induction of ABC-transporter genes. Because ABC-transporter transcriptional induction is supposed to be evaluated in malign tissue, malign transformation of respective tissue in reporter mice needs to be achieved as well. Today, several methods of carcinogen treatment–mediated generation of tumors in experimental animals have been developed and published (Liu et al. 2015). For instance, N-nitroso compounds (an initiator such as N-nitrosodiethylamine) and polycyclic aromatic hydrocarbons (a promoter such as 2-acetylamino-fluorene) will lead to liver tumors, whose *mdr1a* expression can subsequently be monitored by luminescence bioimaging of the liver. However, the background luminescence from benign liver tissue could hinder clear distinguishing *mdr1a* induction in the tumor from *mdr1a* induction in healthy liver cells. Consequently, specific mdr1a.fLUC recombination and tumor generation in tissue distant from the intestine and liver is desirable. For instance, tissue-specific mdr1a.fLUC knock-in in the oral cavity and carcinogen-driven generation of squamous cell carcinoma seems to be a more promising approach. To recombine conditional alleles in murine stratified epithelia of adult animals, Caulin et al. (2004) have developed a system based on the generation of transgenic mice that express a RU486 (progesterose antagonist)–inducible Cre recombinase. This recombinase gene is in turn under the control of the K5 (keratin) promoter, being specific for stratified epithelia of the oral cavity. The inducible features of Cre were achieved by fusing Cre to a deletion mutant of the human progesterone receptor (PR), which fails to bind progesterone but can be activated by RU486. This fusion protein (Cre*PR1) is sequestered in the cytoplasm and translocates to the nucleus after activation with RU486 (Caulin et al. 2004). In the nucleus, Cre*PR1 mediates the excision of LoxP-flanked DNA sequences such as those found in the mdr1a.flox mice. mdr1aflox/wt mice that carry Cre*PR1 in the hemizygous state need to be engineered first. Then, RU486 (e.g., 100 µl of 0.2 µg/µL in sesame oil) can be applied in the oral cavity (Lu et al. 2006) of respective mice to promote recombination to mdr1a.fLUC in the oral cavity selectively. These recombined mice (with some luminescence in the oral cavity) can then be put into a carcinogenesis system: 4-nitroquinoline 1-oxide (4-NQO) is a water-soluble quinoline derivative that can be used to cause tumors in the oral cavity (Liu et al. 2015; Tang et al. 2004). Administration of 4-NQO produces a temporal carcinogenesis progression model demonstrating multiple dysplastic, preneoplastic, and neoplastic lesions after long-term treatment (Hawkins et al. 1994) and thus mimics human tumorigenesis (Rubin et al. 1995; Serewko et al. 2002). Taken together, 4-NQO can be used to generate oral cavity tumors in the reporter mice with mdr1a.fLUC expression in that same tissue. Having established such tumors, mice can be treated with proposed PXR activators (e.g., taxanes) or PXR inherent compounds such as 5-FU (Fig. 3A).

For evaluating *mdr1a* induction, consistent regions of the entire oropharyngeal area should be recorded. This approach can additionally be used to estimate tumor volumes. To distinguish increased *mdr1a* expression “per cell” due to transcriptional induction from increased overall *mdr1a* expression resulting from tumor growth, the mean luminescence intensity should be normalized to a certain surface area (e.g., square inch). An increasing ratio will consequently indicate increased *mdr1a* expression “per cell,” whereas a constant ratio likely demonstrates tumor growth with increased total luminescence (Fig. 3B). In contrast, if there is Darwinian selection of *mdr1a* expressing cancer cells (still having the second *mdr1a* gene to mediate MDR), high-level luminescence will unlikely return to basal levels between the cycles of chemotherapy (Fig. 3B).

In conclusion, with such a reporter gene approach (or variants of), *mdr1a* expression and transcriptional induction can eventually be monitored in vivo, real time, and hardly invasive during carcinogenesis and treatment of tumors. It can also demonstrate the pharmacodynamics (dose–response relationship), sustainability, and contribution of transcriptional induction of *mdr1a* to MDR.

## Concluding remarks

ABC-transporter-mediated MDR remains an attractive scientific topic because both the molecular mechanisms and the actual clinical relevance are still not clear (Robey et al. 2018). Since its first description about five decades ago, P-gp has been investigated in much detail. Many different mechanisms of its regulation have been described. Enhanced transcription of the *ABCB1* gene can result from gene arrangements or epigenetic modifications. While NRs can be important parts of these cellular achievements, the contribution of drug-activated NRs to the dynamic and temporarily restricted induction of *ABCB1*/P-gp and subsequent clinical chemotherapy resistance is poorly understood. One reason might be that most experimental in vitro set-ups use very high drug concentrations. Such high selective pressures in fact led to considerable ABC-transporter overexpression, but unlikely through activation of *ABCB1* regulators such as PXR. Moreover, drug resistance other than transporter overexpression is well known to originate from selection of pre-existing sub-clones with intrinsic drug resistance (Huff et al. 2005, 2006; Gerlinger and Swanton 2010), resembling a scenario known from antibiotic resistance in bacteria (Creager 2007). Thus, it seems nearby to extrapolate to cytotoxic drug treatment of cancer. However, a precise or even quantitative estimation of the respective contributions of Darwinian selection vs. drug-mediated transcriptional induction (mediated by NRs) has never been performed.

Here, an algorithm is suggested that can distinguish between the two simplified scenarios. Sound experimental evidence supports the assumption that in most experimental cases, selection of the “fittest” cells takes place. In contrast, NR-mediated enhanced transcription of transporter genes can be rather regarded as a “first-aid kit” of cancer cell populations for protection against rather low cytotoxic drug concentrations. Later on, non-transcriptional mechanisms (post-translational regulation, enhanced epithelial-to-mesenchymal transition, etc.; Yano et al. 2018) or transcription triggered by genetic or epigenetic events (Chen and Sikic 2012) come to the fore.

For a precise evaluation of the conditions promoting either mode of ABC-transporter overexpression, in vitro and in vivo (mouse reporter gene assays) set-ups are suggested. With this approach, concentrations, drugs, exposure times, or their numerous combinations can be screened for the relevance of transcriptional induction vs. Darwinian selection. Yielding robust, reproducible results, this data can then be translated to clinical evaluations to finally scrutinize “the many ways to turn on P-gp” (Callaghan et al. 2008) or approaches to hinder the development of ABC-transporter-mediated MDR and chemotherapy unresponsiveness.

## Data Availability

Literature and figures are available upon request.

## Abbreviations

MDR: Multidrug resistance
P-gp: P-glycoprotein
MRP: Multidrug resistance–associated protein
PXR: Pregnane-x-receptor
ABC-transporter: ATP-binding cassette transporters
